# Supplementary material for: Piezo1 is a mechanically activated ion channel and mediates pressure induced pancreatitis
Source: Nat Commun. 2018 Apr 30;9:1715. doi: 10.1038/s41467-018-04194-9 (PMC5928090; doi:10.1038/s41467-018-04194-9)
Supplement: Supplementary file 1 — Supplementary information [file 41467_2018_4194_MOESM1_ESM.pdf]

Piezo1 is a mechanically activated ion channel and mediates pressure-induced pancreatitis

Romac, Shahid et al.

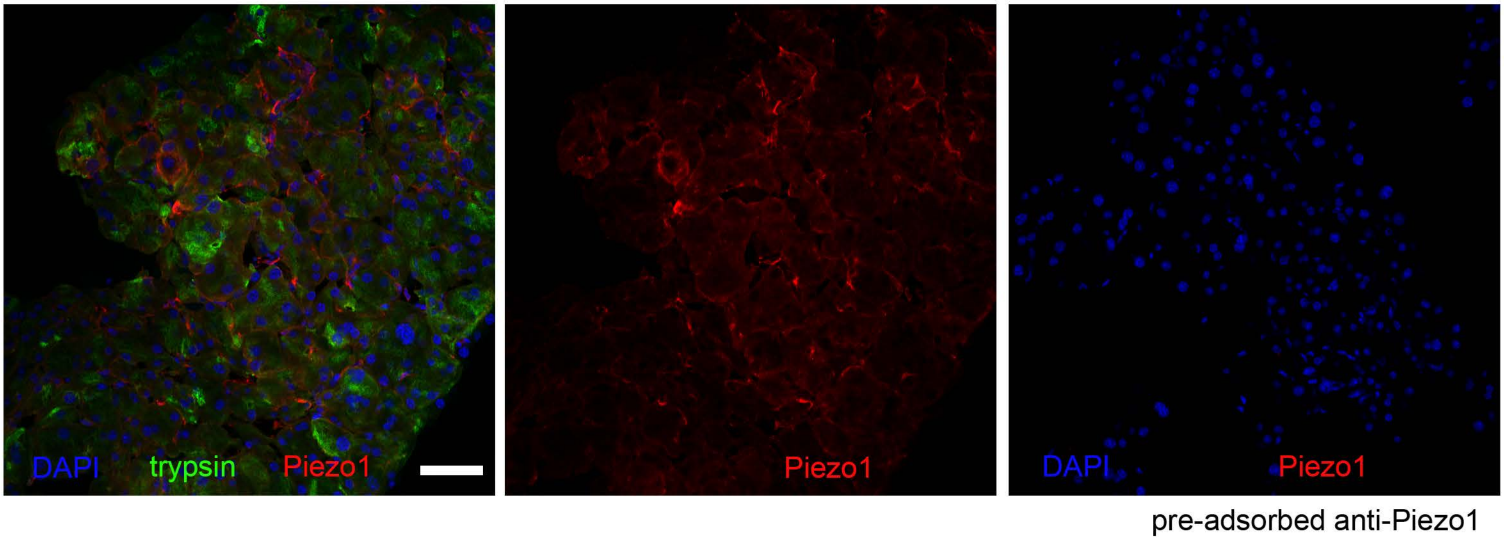

Supplementary Figure 1. Immunostaining of Piezo1 in mouse pancreas. Left panel: immunostaining of trypsin (green) and Piezo1 (red) using a goat anti-trypsin 3 antibody (1/400) and a rabbit anti-Piezo1 antibody (1/300), respectively. DAPI stained nuclei are blue. Middle panel: The same field as the left panel is viewed with the red channel only to illustrate anti-Piezo1 antibody staining. Right panel: Pancreas immunostained with the Piezo1 antibody pre-adsorbed with the immunogenic peptide; note the absence of staining. Images were obtained by confocal microscopy with a 20X objective. Bar = 50 μm.

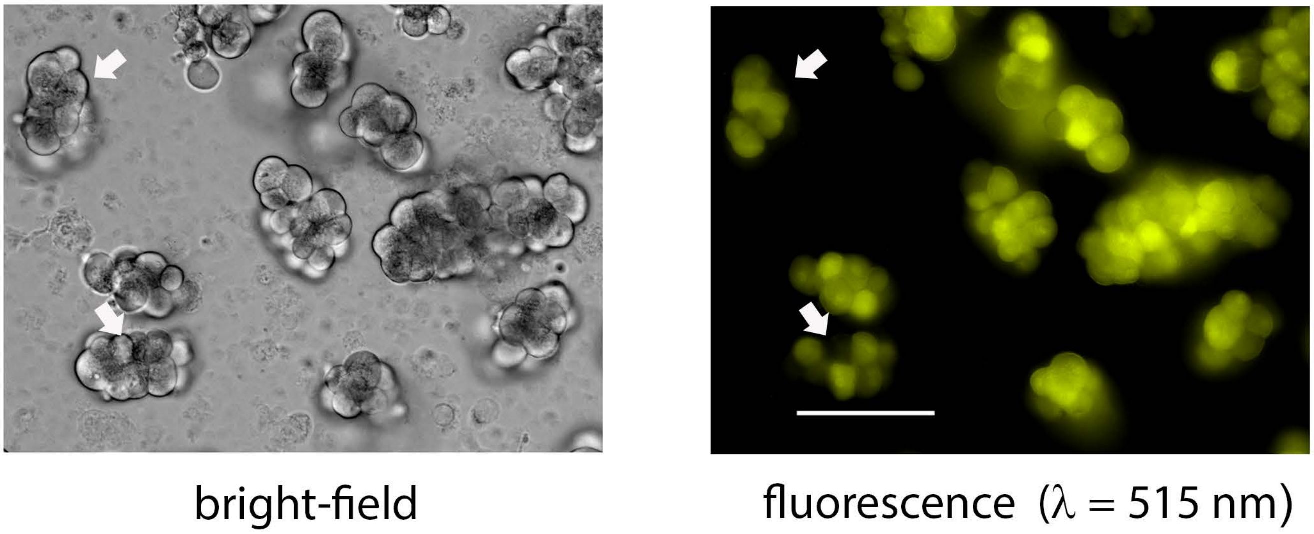

Supplementary Figure 2. Pancreatic acinar cells express Cre after tamoxifen induction. A representative image of isolated acini from Ptf1a;EYFP;Piezo1^{aci}KO mice 8 days after tamoxifen induction. Acini were prepared 8 days after the last tamoxifen injection. Ninety three percent of acinar cells expressed EYFP indicating detectable Cre expression. Arrows indicate the occasional acinar cell that did not express EYFP. The image was taken with a 20X objective. Bar = 100 µm.

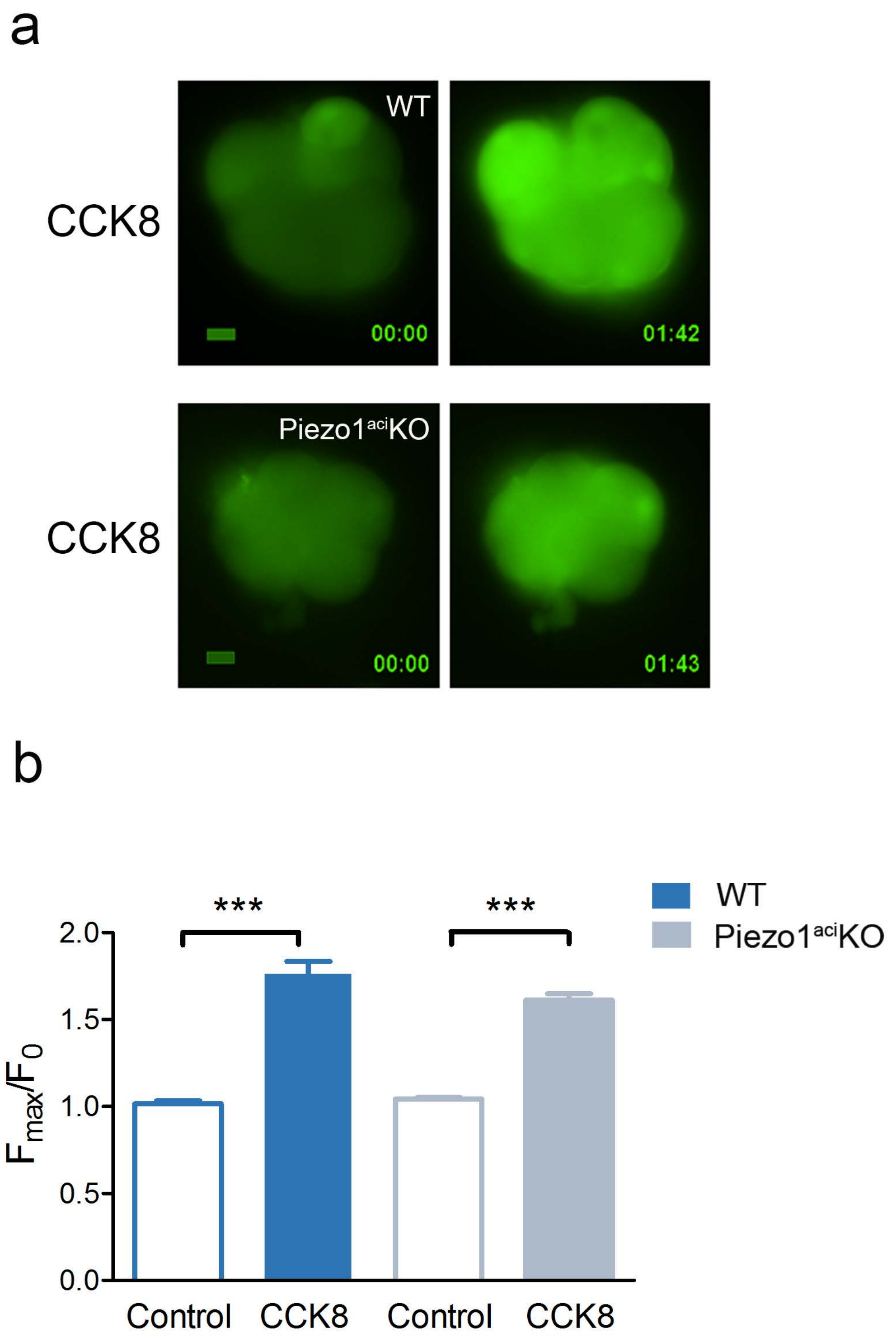

Supplementary Figure 3. Cholecystokinin (CCK8) (20 pM) increases $[Ca^{2+}]_i$ in pancreatic acinar cells of Piezo1^{aci}KO or littermates wild type mice. (**a**) Live cell imaging of pancreatic acini loaded with Calcium 6 stimulated with CCK8 at time 0 (left panel) and at the time (in minutes) of maximum fluorescence (right panel). Bar = 10 μm. (**b**) Peak calcium fluorescence (F_{max}/F_0) is shown (n = 3 experiments; 20 cells per condition). Maximum CCK8-stimulated calcium fluorescence was not different between wild type and Piezo1^{aci} KO mice. ***$P \leq 0.001$.

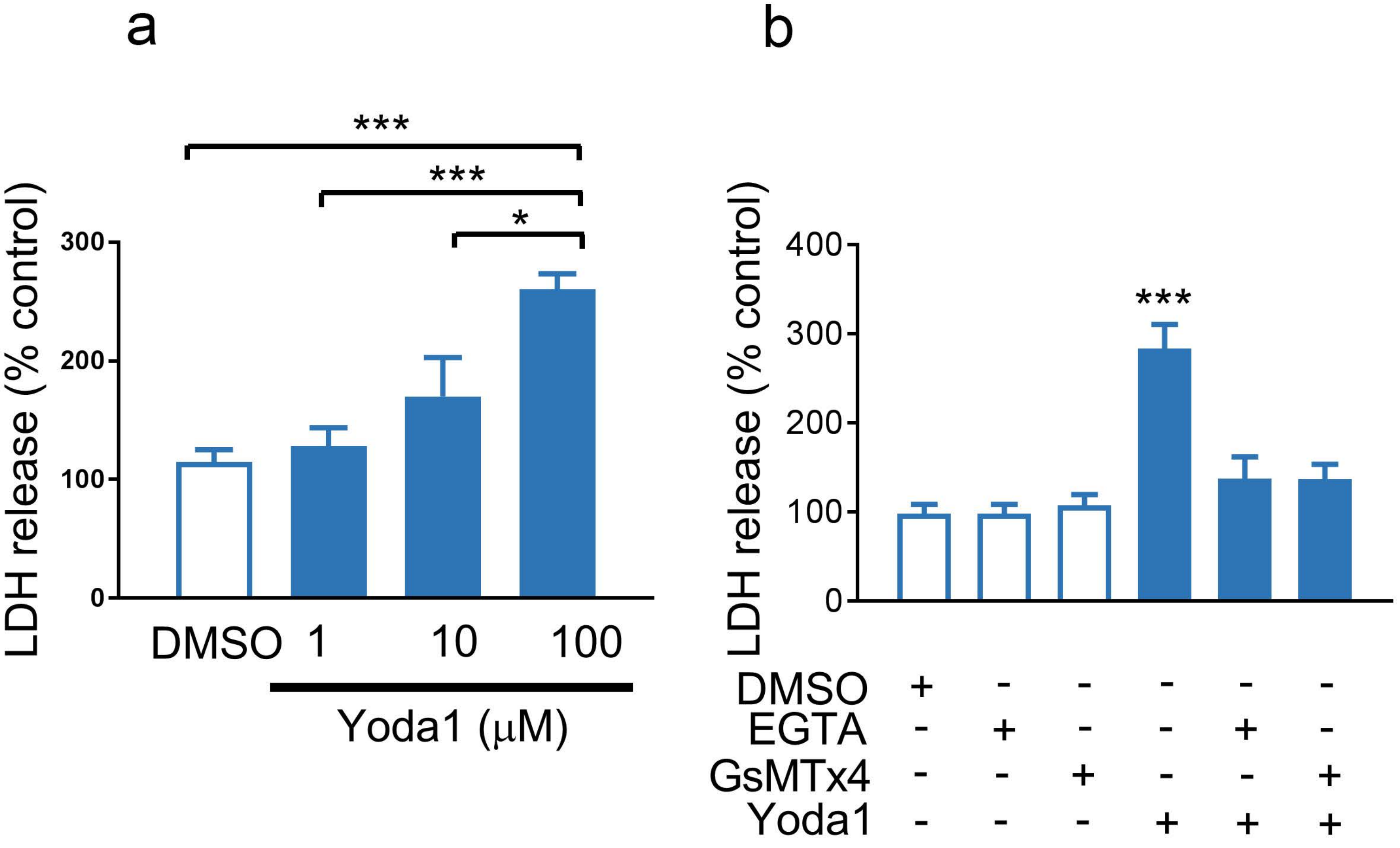

Supplementary Figure 4. The Piezo1 agonist Yoda1 stimulates LDH release from isolated pancreatic acini. Cells were incubated for 30 minute at 37°C. No reagent or chemical were added to the cells in control wells. LDH values in control were used as reference. (**a**) LDH released into the media of acini from C57BL/6J mice following stimulation with Yoda1 (n = 4-5). (**b**) LDH release from C57BL/6J acini following Yoda1 (100 μM) stimulation in the presence of GsMTx4 (2.5 μM) or in D Tris buffer with added EGTA (2 mM) (n = 6). * *P* ≤0.05, *** *P* ≤0.001. Measurements were performed after 30 minute incubation at 37°C.

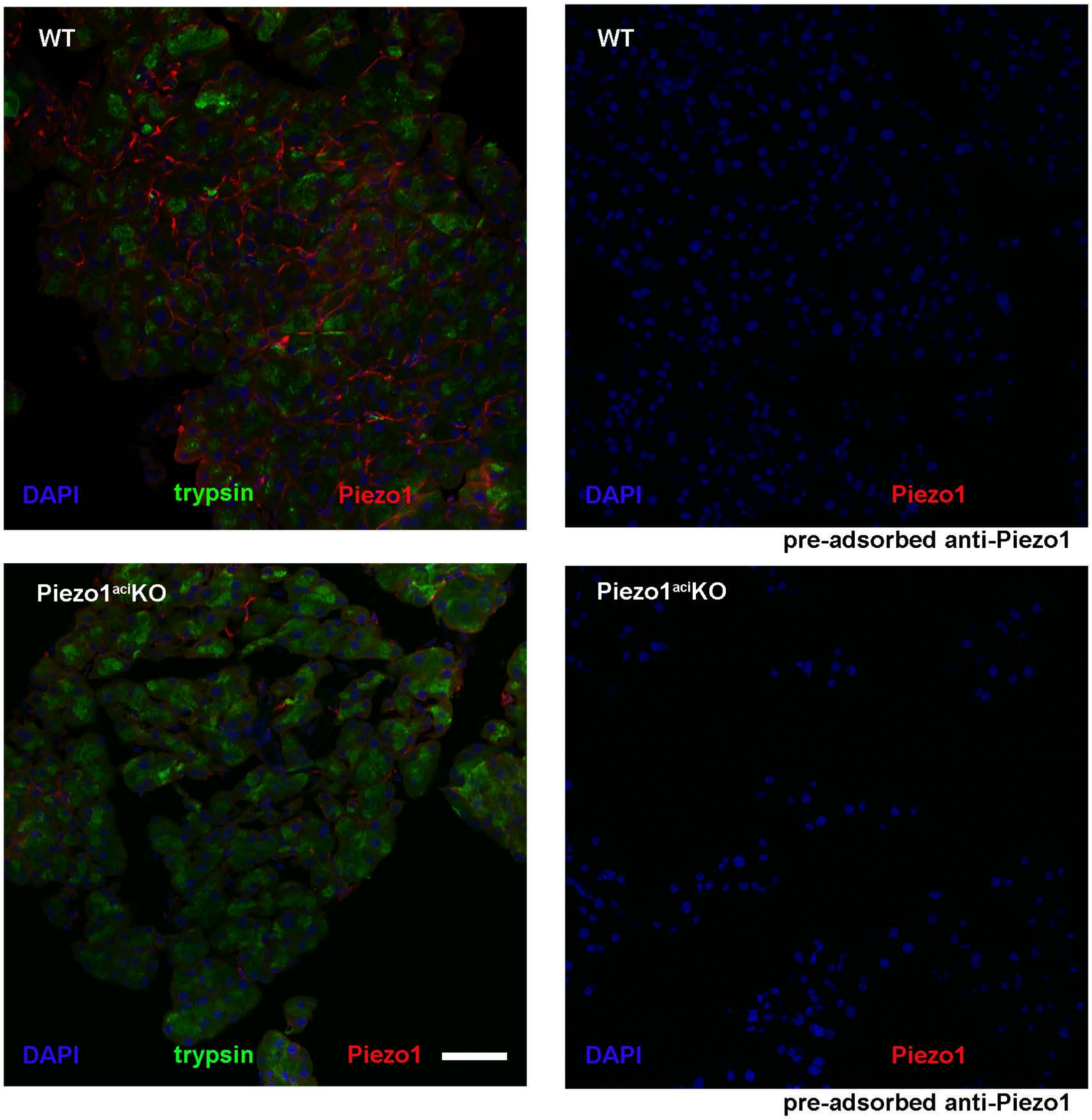

Supplementary Figure 5. Immunostaining of Piezo1 in pancreas. Left panel: immunostaining of trypsin (green) and Piezo1 (red) using a goat anti-trypsin 3 antibody (1/400) and a rabbit anti-Piezo1 antibody (1/300), respectively. DAPI stained nuclei are blue. Pancreas from a littermate wild type mouse is shown in the upper row and pancreas from a Piezo1^{aci}KO mouse is shown on the lower row. Right panel: immunostaining with a rabbit anti-Piezo1 antibody (1/300) that was pre-adsorbed with its immunogenic peptide. Images were obtained by confocal microscopy with a 20X objective. Bar = 50 μm.

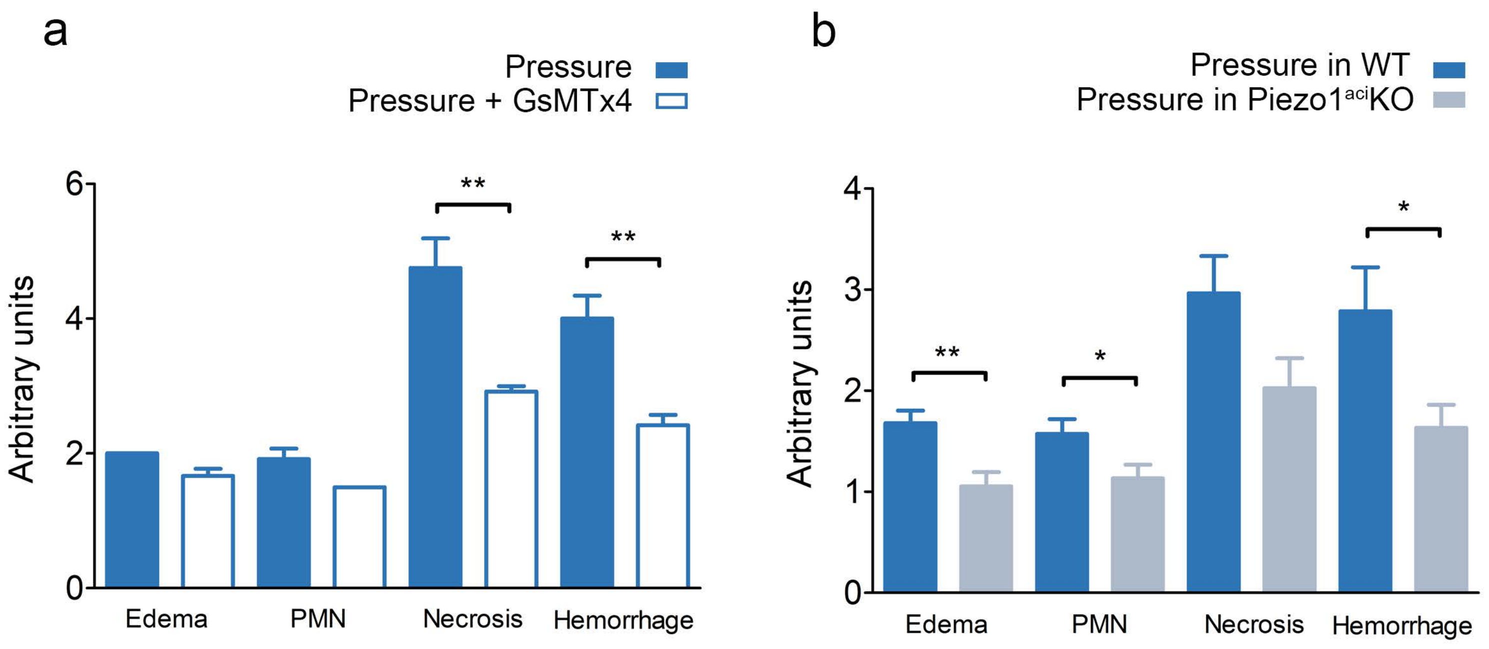

Supplementary Figure 6. Analysis of individual parameters for histological scoring. (**a**) Pressure experiments using C57BL/6J mice described in Figure 3. (**b**) Pressure experiments with WT littermates and Piezo1^{aci}KO mice reported in Figure 4.

Edema was scored using a scale of 0 to 3 with 0.5 increments: none is considered 0, mild is 1, moderate is 2 and severe is 3. Neutrophil (PMN) infiltration was scored on a scale of 0 to 3 with 0.5 increments: none is 0, mild is 1, moderate is 2 and severe is 3. Necrosis was scored on a scale of 0 to 7 with increments of 0.5. We measured only the parenchymal necrosis using the following criteria: none is 0; focal, mild is up to 3; and/or sublobular, moderate is up to 5; and/or lobular is up to 7. Hemorrhage was scored also on a scale of 0 to 7 with increments of 0.5. None is 0, mild is up to 3, moderate is up to 5 and severe is up to 7. Tabulation of all scores can yield a maximum score of 20. Values between 2 groups were compared using Unpaired Student's t test. Mean +/- SEM. * = $P < 0.05$, ** = $P < 0.01$.
