# Supplementary material for: Piezo1 is a mechanically activated ion channel and mediates pressure induced pancreatitis
Source: Nat Commun. 2018 Apr 30;9:1715. doi: 10.1038/s41467-018-04194-9 (PMC5928090; doi:10.1038/s41467-018-04194-9)
Supplement: Supplementary file 3 — Description of Additional Supplementary Files [file 41467_2018_4194_MOESM3_ESM.pdf]

Description of Additional Supplementary Files

File Name: Supplementary Movie 1
Description: Piezo1 agonist Yoda1 induces intracellular calcium rise in WT pancreatic acini.

File Name: Supplementary Movie 2
Description: GsMTx4 blocks Yoda1 induced intracellular calcium rise in WT pancreatic acini.

File Name: Supplementary Movie 3
Description: Yoda1 fails to induce intracellular calcium rise in Piezo1^{aci}KO pancreatic acini.
